# Three-dimensional Organotypic Culture Models of Human Hepatocellular Carcinoma

**DOI:** 10.1038/srep21174

**Published:** 2016-02-16

**Authors:** Atsushi Takai, Valerie Fako, Hien Dang, Marshonna Forgues, Zhipeng Yu, Anuradha Budhu, Xin Wei Wang

**Affiliations:** 1 Laboratory of Human Carcinogenesis, Center for Cancer Research, National Cancer Institute, Bethesda, Maryland 20892, USA

## Abstract

Three-dimensional cell culture methods are viable *in vitro* approaches that facilitate the examination of biological features cancer cells present *in vivo*. In this study, we demonstrate that hepatocellular carcinoma (HCC) cells in porous alginate scaffolds can generate organoid-like spheroids that mimic numerous features of glandular epithelium *in vivo*, such as acinar morphogenesis and apical expression patterns of EpCAM, a hepatic stem/progenitor cell marker highly expressed in a subset of HCC with stemness features. We show that the activation of Wnt/β-catenin signaling, an essential pathway for maintaining HCC stemness, is required for EpCAM^+^ HCC spheroid formation as well as the maintenance of the acinous structure. Furthermore, we demonstrate that EpCAM^+^ HCC cells cultured as spheroids are more sensitive to TGF/β-induced epithelial-mesenchymal transition with highly tumorigenic and metastatic potential *in vivo* compared to conventional two-dimensional (2D) culture. In addition, HCC cells in EpCAM^+^ spheroids are more resistant to chemotherapeutic agents than 2D-cultured cells. The alginate scaffold-based organotypic culture system is a promising, reliable, and easy system that can be configured into a high throughput fashion for the identification of critical signaling pathways and screening of molecular drug targets specific for HCC.

Hepatocellular carcinoma (HCC), a major histological subtype of liver cancer, represents a lethal form of solid tumors with at least 600,000 deaths in the world annually^1^ and a reported 5-year survival rate of <20% in the US^2^. The main reason that many patients are refractory to treatment and have dismal outcome is the extended biological heterogeneity observed in HCC^3^. Such tumor heterogeneity makes it difficult to identify specific druggable cancer driver genes whose functions are essential for the fitness of cancer cells. As such, consensus driver targets, which act as the ‘Achilles heel’ of cancer, a phenomenon known as oncogene addiction^4^, are not available for HCC therapeutic intervention. This may contribute to the recent major setback for evaluating molecularly targeted agents^5^. Identifying and molecularly targeting key driver genes specific for a particular subgroup of HCC may be the key to improving the current therapeutic status. Recent global cancer genomic studies have allowed for the identification of many candidate driver genes^6^,^7^. However, each tumor appears to carry numerous genomic alterations with significant heterogeneity amongst each other. The presence of considerable genomic alterations constitutes a bottleneck to effectively rank, triage and evaluate these candidate driver genes as druggable targets. Thus, there is an urgent need to develop a simple and pathophysiologically-relevant model to efficiently evaluate candidate drivers.

Pre-clinical research to delineate molecular mechanisms that drive cancer growth and progression is usually carried out in two-dimensional (2D) cell culture systems, which are efficient and reliable, but lack the appropriate cell-cell contact environment typically observed *in vivo*. Although genetically-modified animals are used as an alternative to overcome the limitation of 2D cell culture^8^,^9^, they are costly and time-consuming experiments to conduct. Furthermore, there is a considerable difference between human cells and animal cells with regard to the requirements for oncogenic transformation and immune surveillance. It has been demonstrated that three-dimensional (3D) organotypic human cancer cell models as well as hepatocyte spheroid models are viable alternatives^10^,^11^,^12^, as they can be tailored to be biomimetic and accurately recapitulate the native *in vivo* scenario. For example, rat hepatocytes in 3D cultures possess structural polarity and channels with great similarity in structure and function to bile canaliculi, which can explain their enhanced hepatocellular activities^12^,^13^,^14^. In addition, Lgr5^+^ mouse liver stem cells can be expanded as transplantable organoids that retain many characteristics of the original epithelial architecture^15^. In contrast to normal cells, tumor cells with stem cell features such as EpCAM^+^ human HCC cells, can also generate 3D spheroids^16^. Thus, the 3D organotypic model provides an important alternative to both 2D culture and *in vivo* animal model systems.

Here, we describe the characterization of an AlgiMatrix-based 3D culture method to support HCC organoid formation. Using this method, we demonstrate that certain EpCAM^+^ HCC cells can generate organoid-like spheroids that recapitulate numerous features of the glandular epithelium *in vivo*, such as formation of acini and apical expression patterns of stem cell-associated proteins. We show that β-catenin signaling, including EpCAM that is essential for maintaining HCC stemness^16^,^17^,^18^, is required for the formation of EpCAM^+^ HCC organoids. Moreover, we show that compared to cells in 2D culture, EpCAM^+^ HCC organoids are resistant to chemotherapeutic agents, but are sensitive to TGF-β-induced epithelial-mesenchymal transition (EMT) and are highly tumorigenic and metastatic *in vivo*. We suggest that the AlgiMatrix-based HCC organoid culture system represents a reliable and efficient *in vitro* model for investigating candidate HCC driver genes and molecularly-targeted drug screening.

## Results

### AlgiMatrix-based 3D culture

To investigate whether HCC cells can form organoid-like spheroids resembling features of the glandular epithelium *in vivo*, such as undergoing acinar morphogenesis, we compared 6 hepatoma cell lines using several matrices and methodologies. These cells were chosen for initial testing since they were previously fully characterized for stemness features and stem cell marker expression^16^. Specifically, we examined Huh1 hepatoma cells^19^ and Huh7 HCC cells^20^, both of which contain a heterogeneous population of EpCAM^+^ and EpCAM^−^ cells. We also examined HepG2 HCC cells and Hep3B HCC cells^21^. HepG2 and Hep3B cell lines contain homogenous populations of EpCAM^+^ cells. Finally, we examined homogenous EpCAM^−^ cell lines SK-Hep-1 tumorigenic endothelial-like liver adenocarcinoma and MHCC97 HCC cells^22^,^23^.

To determine the optimal matrix for the growth of organoid-like spheroids, we compared various matrices including AlgiMatrix, Matrigel and GigaMatrix, to the commonly used Ultra-Low attachment surface for supporting HCC spheroids. We examined several quantitative and qualitative factors including sphere size, sphere morphology, possibility of sphere fusion, and the ease in which intact spheres could be collected for further characterization and use. We first chose to eliminate GigaMatrix, because we observed irregular shaped spheroids in addition to round spheroids, as well as instances of sphere fusion (Suppl Table 1, Suppl Fig. 1). In addition, we found that isolating spheres from the GigaMatrix material was difficult in nature. We next eliminated Matrigel because we found the formation of irregular spheres, as well as many instances of sphere fusion. We found that with Ultra-Low attachment plates, spheres were very round and showed no instances of fusion, but could be found in a wide size range (20–300 μm). In contrast, in AlgiMatrix we observed very regular, round sphere morphology and no sphere fusion (Suppl Fig. 1). We also found that spheres in AlgiMatrix could be collected very easily for additional studies, such as H&E staining for morphology. Taking into consideration each of the factors mentioned above, we chose AlgiMatrix in favor of Ultra-Low attachment plates for further evaluation in order to recapitulate an *in vivo*-like environment with extracellular matrix.

**Figure 1:**
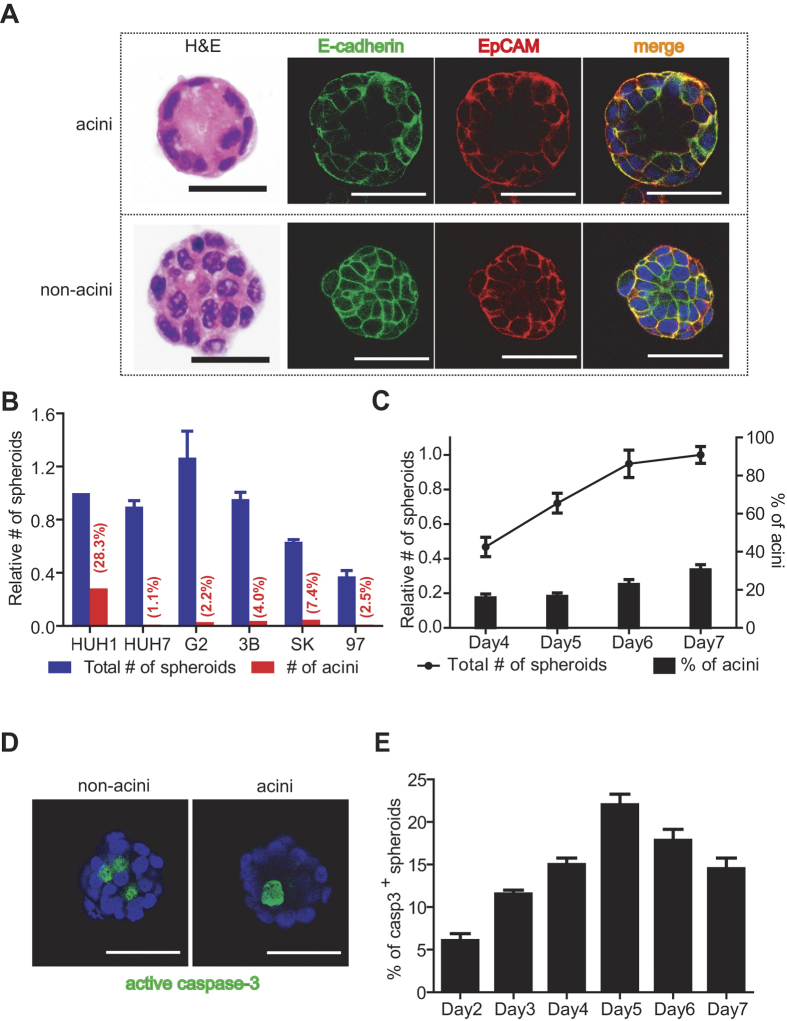
(**A**) Representative images of Day 7 Huh1 acini (top) and non-acini (bottom). Shown are spheres stained with H&E, and immunostained for E-cadherin (green) and EpCAM (red). Scale bars are 50 μm. (**B**) Relative number of spheres at Day 7 in each cell line is shown. Blue bars and red bars indicate relative number of total spheres and acini, respectively, based on the total number of Huh1 spheres. The percentage value in parentheses shown above each red bar indicates the ratio of acini to total spheres. Total numbers of spheroids are presented as the average of five independent experiments, whereas numbers of acini are presented as the average of duplicate experiments. (**C**) Relative number of Huh1 spheres and the proportion of acini in noted culture periods are shown. The line plot indicates relative number of total spheres based on the sphere number at Day 7. Each bar indicates the proportion of acini in total spheres. Data are presented as the average of at least three independent analyses. (**D**) Representative images of spheres immunostained for active caspase-3 (green) are shown. (**E**) The proportion of active caspase-3 positive spheres in total spheres is shown. Data are presented as the average of at least three independent analyses. All error bars shown represent ±SEM.

To examine the morphological appearance of cell spheroids in AlgiMatrix scaffold, we compared the 6 human HCC cell lines described above (i.e., Huh1, Huh7, HepG2, Hep3B, SK-Hep-1, and MHCC97), as well as an immortalized human normal hepatocyte line HHT4, and cultured human primary hepatocytes. All HCC cell lines formed spheroids with varying degrees in size and shape as analyzed by H&E staining (Suppl Fig. 2). In contrast, HHT4 and primary hepatocytes do not form spheroids under this condition, nor do they form spheroids in any of the other matrices tested (data not shown), suggesting that these cells likely do not have a capacity for sphere formation. Among all HCC cell lines cultured for 7 days in AlgiMatrix, some spheroids contain a lumen (Fig. 1A). Interestingly, while all Huh1 spheroids were positive for EpCAM, EpCAM^+^ cells were mainly localized in the outer layers of spheroids with an apical-basal like expression pattern (Fig. 1A). This expression pattern was not evident with E-cadherin (Fig. 1A). Thus, a polarized expression pattern appeared unique to EpCAM.

Most importantly, we observed that spheroids with a ring-shape ‘acinus’ glandular-epithelial-like appearance could be found in every HCC cell line examined when cultured in AlgiMatrix, indicating that AlgiMatrix can support the formation of glandular architecture, thus confirming our choice of AlgiMatrix as a useful matrix for organoid growth. Interestingly, acini formation was most frequently found in Huh1 (Fig. 1B). Approximately 28.3% of Huh1 spheroids contained acini at Day 7, compared to less than 10% of other HCC cell lines. An increase in the numbers of all spheroids and acini spheroids were evident when culturing between Day 4 and Day 7 (Fig. 1C). Since acini spheroids may be the result of apoptosis of cells in the luminal space during acinar morphogenesis^24^, we determined the status of activated caspase-3 in Huh1 spheroids. We found that caspase-3^+^ cells were mainly located inside but not at the surface of the spheroids, as determined by confocal microscopy analysis (Fig. 1D). An increase in the numbers of caspase-3^+^ spheroids was evident during a culture period between Day 2 and Day 5 (Fig. 1E). Due to the intrinsic propensity of Huh1 cells for glandular-like acini formation, we chose Huh1 cells as the model cell line for further studies.

### Molecular signaling in HCC Spheroids

**Figure 2:**
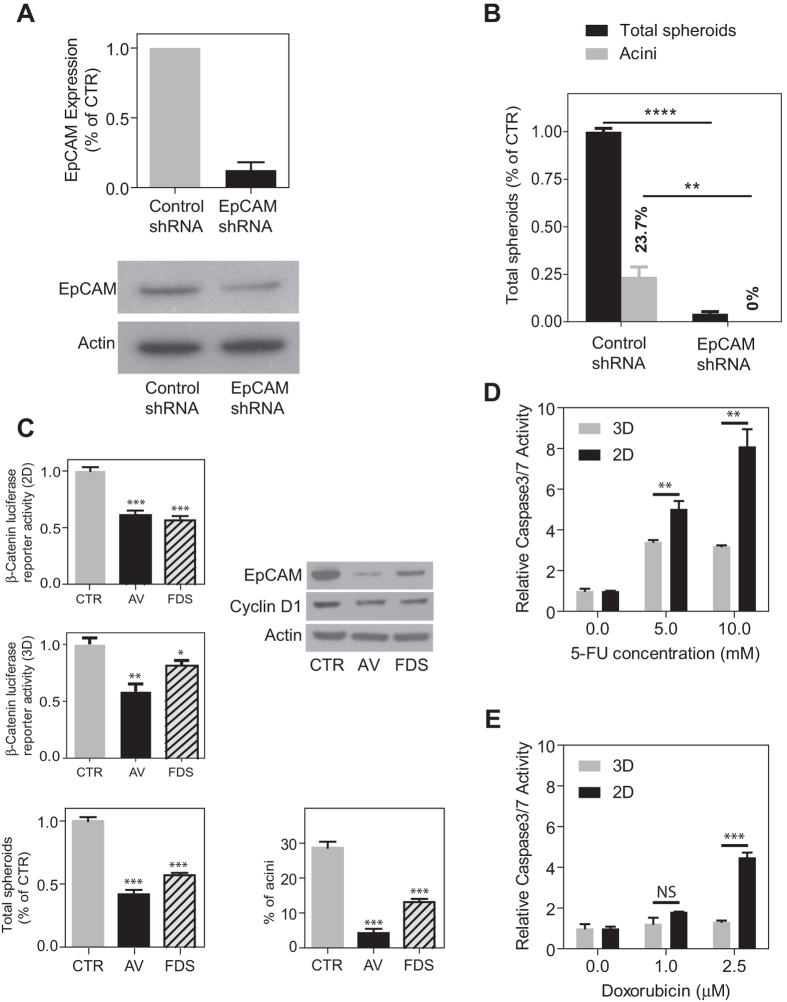
(**A**) EpCAM expression level in Huh1 cells treated with control shRNA or EpCAM shRNA were determined by quantitative RT-PCR (top panel) and western blotting (bottom panel). RT-PCR data are presented as the average of four independent readings. (**B**) Relative number of Huh1 spheres treated with control shRNA or EpCAM shRNA is shown. Black bars and grey bars indicate relative number of total spheres and acini, respectively, based on the total number of control spheres. The percentage value shown above each grey bar indicates the ratio of acini to total spheres. Data are presented as the average of triplicate experiments. (**C**) β-catenin luciferase reporter activity in 2D cells (upper left panel) and 3D spheroids (middle left panel), EpCAM and Cyclin D1 expression level in 3D spheroids (upper right panel), total number of spheres (bottom left panel) and the proportion of acini (bottom right panel) treated with DMSO, AV-606 (AV, 3 μM) and fiduxosin (FDS, 10 μM) are shown. Data are presented as the average of at least three independent experiments. (**D**,**E**) Relative caspase 3/7 activity of 2D or 3D-cultured Huh1 cells treated with anti-cancer drugs (D, 5-FU; E, doxorubicin). Data are presented as the average of three independent analyses. All error bars shown represent ±SEM. **p < 0.01, ***p < 0.001.

Since EpCAM is an HCC stemness biomarker and plays a critical role in tumorigenicity of EpCAM^+^ HCC cells^16^,^17^, and because all Huh1 spheroids were positive for EpCAM (Fig. 1A), we determined whether EpCAM is necessary for HCC spheroid formation. Using an EpCAM-shRNA lentiviral vector, EpCAM expression was efficiently inhibited in Huh1 cells (Fig. 2A). In parallel, a significant reduction in the numbers of all spheroids and acini spheroids was evident upon EpCAM silencing, however no morphological changes in spheres following EpCAM silencing was noted (Fig. 2B, Suppl Fig. 3). Because EpCAM is a direct transcriptional target of Wnt/β-catenin signaling^17^, we also examined whether β-catenin inhibitors have an effect on Huh1 spheroid formation using small molecular inhibitors of wnt/β-catenin signaling that were previously identified via a high throughput screen (i.e., AV606 and fiduxosin^25^). The optimum concentration of each drug was determined by adopting a dose in which the number of Huh1 spheroids formed (Wnt/β-catenin active) is at least 1.5-fold less than the number of MHCC97 spheroids formed (Wnt/β-catenin negative) (Suppl Fig. 4). Consistently, these compounds significantly inhibited β-catenin luciferase reporter activities in 2D culture and 3D spheroids, as well as the expression of direct wnt targets EpCAM and Cyclin D1 in 3D spheroids (Fig. 2C). The formation of total spheroids and acini spheroids of Huh1 cells was significantly inhibited (Fig. 2C). We also determined if the cells comprising the spheroids are sensitive to conventional chemotherapeutic drugs such as 5-Fluorouracil (5-FU) and doxorubicin. We found that Huh1 spheroids (3D) were more resistant to 5-FU and doxorubicin-induced apoptosis than cells in monolayer culture (2D) as determined by caspase3/7 activity (Fig. 2D, E).

### HCC organoids and epithelial-mesenchymal transition

**Figure 3:**
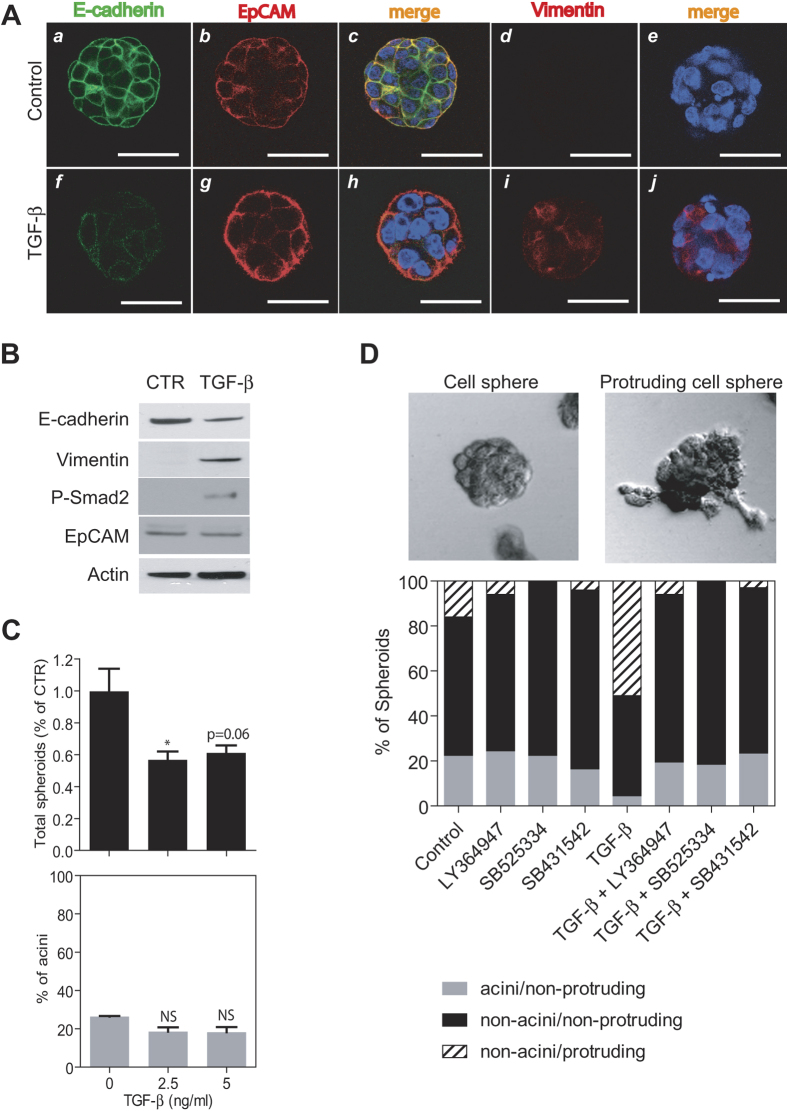
(**A**) Representative images of Huh1 spheres at Day 7 with no treatment (top panels) and 2.5 ng/ml of TGF-β added at day 5 of spheroid culture (bottom panels). Shown are spheres immunostained for E-cadherin (a,f; green), EpCAM (b,g; red) and merged (c,h). Other representative spheres immunostained for vimentin (d,i; red) and merged (e,j) are also shown. Scale bars are 50 μm. (**B**) The expression level of E-cadherin, vimentin, phosphorylated Smad2 and EpCAM in Huh1 spheres treated with 5 ng/ml TGF-β at day 3 of spheroid culture. (**C**) Relative number (black bar) and the acini proportion (grey bar) of Huh1 spheres in the presence of TGF-β are shown. Data are presented as the average of three independent analyses. (**D**) Representative images of round-shape cell spheres (top, left) and protruding cell spheres (top, right) are shown. The proportion of each sphere type following treatment with 5 ng/ml TGF-β combined with several kinds of TGF-β receptor kinase inhibitors are shown as a bar graph (bottom). Data are presented as the average of duplicate experiments. All error bars represent ±SEM. *p < 0.05.

Stem/progenitor cells retain incredible plasticity that includes their abilities to self-renew and differentiate. They may lose their typical epithelial features and change to a mesenchymal state by activating epithelial mesenchymal transition (EMT) during embryonic and adult glandular development as well as cancer development^26^. Multiple studies have established a critical role of TGF-β-induced EMT in cancer progression, including HCC^27^,^28^. To explore the cell-cell contact environment in HCC spheroids, and to understand how spheroids respond to EMT stimulus, we used TGF-β as a tool to induce EMT in 3D cells, and evaluated spheroid formation. Treatment of Huh1 spheroids with TGF-β in AlgiMatrix culture resulted in a reduced expression of epithelial marker E-cadherin and an increased expression of mesenchymal marker vimentin, indicating induction of EMT in the spheroids (Fig. 3A,B). Phosphorylated Smad2, which is one of the signal mediators induced upon activation of the TGF-β pathway, was detected following TGF-β treatment, confirming pathway activation (Fig. 3B). We also noted that TGF-β treatment had a minimum effect on EpCAM expression (Fig. 3A,B). Under these conditions, TGF-β significantly inhibited the total number of spheroids formed, but not acinar morphogenesis (Fig. 3C). Total spheroid formation was almost completely abrogated when TGF-β was added at the initiation of AlgiMatrix culture on Day 1 (Suppl Fig. 5). In addition, we used Cultrex Spheroid Invasion Matrix to determine the role of TGF-β on cellular invasiveness. We found that TGF-β treatment resulted in an increase in cells migrating out of the spheroids, i.e., protruding cell sphere (Fig. 3D). Strikingly, none of the protruding cell spheroids induced by TGF-β had an acinar morphology. Approximately 50% of protruding cell spheroids were found with TGF-β treatment as compared to 16% in untreated spheroids (Fig. 3D). TGF-β treatment also resulted in a reduction of acini spheroids. Moreover, TGF-β−induced protruding cell spheres could be effectively blocked by three TGF-β receptor kinase inhibitors, i.e., LY364947, SB525334 and SB431542 (Fig. 3D).

### HCC spheroids and tumorigeneity

**Figure 4:**
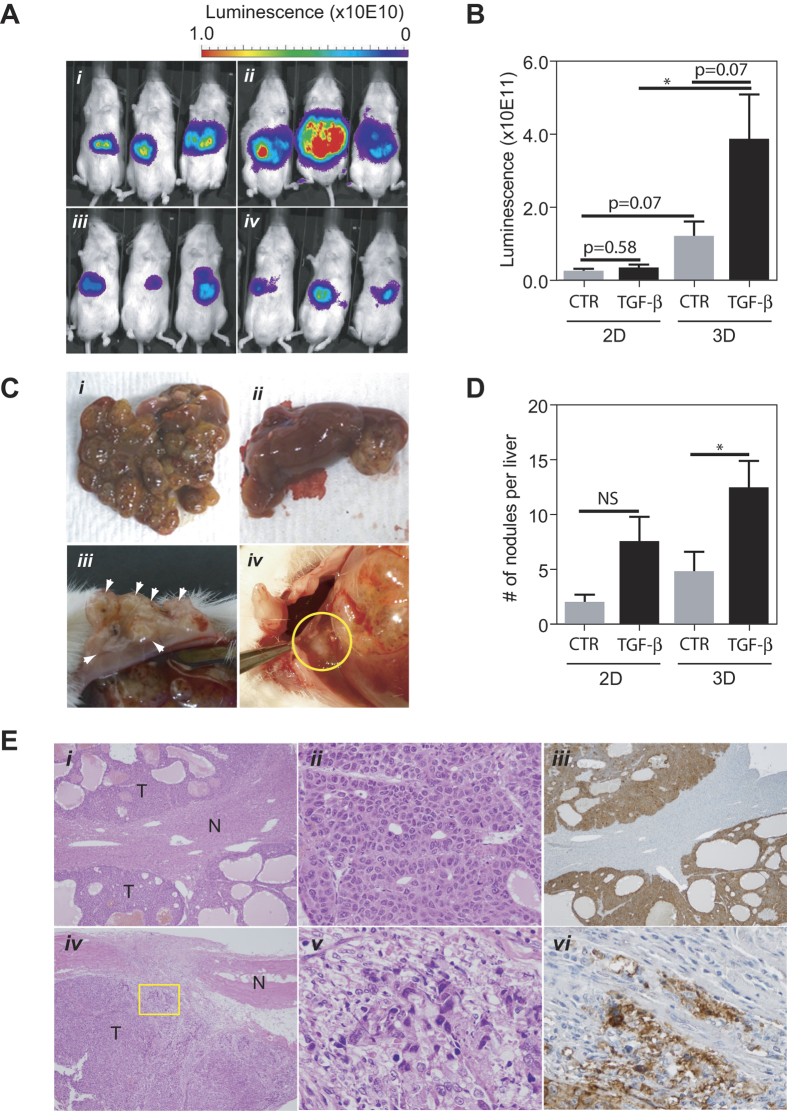
(**A**) Representative whole body luminofluorescence images of mice injected with (i) non-treated spheres, (ii) TGF-β treated spheres, (iii) non-treated cells and (iv) TGF-β treated cells 4 weeks after the surgery. (**B**) Quantification of the luciferase signal in each group is shown. (**C**) Macroscopic tumors developed in the orthotopic liver cancer mouse model. Representative images of mouse liver injected with (i) TGF-β treated spheres and (ii) non-treated cells are shown. The images of metastatic nodule detected in peritoneum (iii, arrow heads) and diaphragm (iv, in the circle) are also shown. (**D**) The average number of macroscopic nodules per liver in each group (right panel) is shown. (**E**) Microscopic images of tumors developed in a mouse injected with TGF-β treated spheres. Top panels (i–iii): liver tumors stained with H&E (i: 40× ii: 200× magnification) and immunostained for HLA-A (iii, 40× magnification). Bottom panels (iv–vi): peritoneal metastasis in the identical mouse stained with H&E (iv: 40× v: 400× magnification) and immunostained for HLA-A (vi, 400× magnification). N and T indicate non-tumor and tumor, respectively. All error bars represent ±SEM. *p < 0.05.

We used an orthotopic liver cancer mouse model^29^ to determine the tumorigenic activities of HCC spheroids treated with or without TGF-β. We established an Huh1 cell line by stably expressing a firefly luciferase reporter that could be easily monitored for tumor growth *in vivo* (data not shown). This experiment included 4 groups; Huh1 cells in 3D culture treated with or without TGF-β, and Huh1 cells in 2D culture treated with or without TGF-β, with 10 animals per group. Only mice that survived the orthotopic surgical procedure were included for further analysis (Suppl Table 2). Using this system, we found that at 4 weeks after HCC cell transplantation, tumor sizes, determined by an *in vivo* image analysis of the luciferase signals, from 3D cultured Huh1 cells were larger than that of 2D cultured cells (Fig. 4A). The luciferase signals were elevated in 3D cells compared to 2D cells, which was further enhanced by TGF-β treatment (Fig. 4A,B). In contrast, TGF-β had no effect on the tumorigenicity of 2D cells (Fig. 4B). Histological analysis revealed that while the frequency of HCC occurrence and the formation of visible tumors in the liver was similar in each treatment group, (Fig. 4C, panels i and ii; Suppl Table 2), the numbers of macroscopic nodules detected in the liver was significantly higher in mice from 3D cells treated with TGF-β, whereas TGF-β had a less significant effect on the number of nodules detected in tumors from 2D cells (Fig. 4C, panel i vs. panel ii, Fig. 4D). Some animals implanted with 3D cells treated with TGF-β developed metastasis into the peritoneum or diaphragm (Fig. 4C, panels iii and iv). In contrast, no visible metastatic tumors were found in other groups. Histologically, all tumors in the liver displayed a trabecular pattern representing typical HCC features (Fig. 4E, panels i–iii). All of the hepatic nodules examined had a clear margin and thus we did not further examine if any invasive cells were visible in the normal liver tissue. In contrast, metastatic nodules in the peritoneum showed different histological characteristics (Fig. E, panels iv–vi). Tumor margins were disrupted and spindle-shaped cancer cells were visible in the normal fibrotic tissue (Fig. 4E). These data suggest that TGF-β-induced-EMT has the potential to transform cancer cells more aggressively by reprogramming cell features *in vivo*, especially in organotypically cultured spheroids.

## Discussion

It has been long recognized that epithelium-derived cells such as MCF-10A cells, which was originally derived from normal mammary glands, can form well-ordered architecture when cultured in appropriate matrices *in vitro.* This model is useful to study oncogene-induced mammary tumorigenicity and tumor invasiveness^10^,^24^. In addition, functional studies of tissue stem cells has been facilitated in recent years by the development of *in vitro* organoid cultures that mimic 3D organ-buds grown *in vivo*^30^. A number of laboratories have successfully generated various organoids^31^,^32^,^33^,^34^. Most recently, cancer cell-derived organoid models from prostate, lung and pancreatic cancers have also been established as an *in vitro* system to model an *in vivo* tumor pathophysiological state, such as tumor-associated signaling pathways and chemoresistance^35^,^36^. However, no reliable HCC organoid models are available at the present time. In this study, we have developed an HCC organoid model based on AlgiMatrix formula to support EpCAM^+^ stem cell-like HCC cell propagation. EpCAM^+^ HCC cells, particularly Huh1 cells, can efficiently form organoid-like spheroids with features of glandular epithelium *in vitro*, which include acinar morphogenesis, but are chemoresistant and have elevated metastases in orthotopic liver cancer models. We also found that EpCAM^+^ HCC cell organoids rely on the presence of EpCAM and β-catenin signaling since RNAi-mediated silencing of EpCAM or small molecular inhibitors of wnt-β-catenin signaling, identified from a high-throughput screen of small molecule libraries from the Developmental Therapeutics Program of NCI^25^, can effectively inhibit organoid formation. Further characterization of these wnt-β-catenin inhibitors, including examining a combination of these inhibitors with conventional chemotherapeutics, using this organotypic culture model is an important future endeavor.

In addition to EpCAM, a number of other biomarkers, such as CD133, CD44, CD24 and CD13, have been reported as biomarkers of HCC cancer stem cells, and it would be highly interesting to characterize cultured 3D spheroids for these stem cell markers in future studies. Furthermore, a variety of signaling pathways contribute to the maintenance of stem-like HCC cells that are positive for these markers^37^. We suggest that this model may allow us to effectively test the pathophysiological roles of various cancer stem cell biomarkers or candidate driver genes identified by global genome-based investigations on tumor initiation and progression, and to achieve rapid assessment of potential pharmacologic inhibitors of HCC.

EMT is a physiological biologic process whereby features of polarized epithelial cells biologically change to a mesenchymal phenotype, such as enhanced ability for cell migration and cell invasion^26^. Especially during the developmental period, the conversion between epithelial cells and mesenchymal cells is critical for organogenesis. On the other hand, EMT also occurs under pathological conditions, such as tissue fibrosis and cancer progression. The significance of EMT in cancer development has particularly been exploited during the metastatic process. A number of pathways which are involved in EMT have been detected in a variety of tumors. TGF-β is a key member of the TGF-β superfamily that induces EMT through both canonical (Smad2/3 dependent) and non-canonical (Smad2/3 independent) signaling inputs^27^. In this study, we have explored the roles of EMT in HCC organoid formation by inducing EMT with TGF-β. We found that TGF-β could inhibit HCC spheroid formation accompanied by a decreased expression of E-cadherin and an increased expression of vimentin, a hallmark of EMT. The blockage of the phenotypic changes by TGF-β receptor kinase inhibitors indicates that TGF-β has a critical role for EMT in HCC spheroids. Further characterization of the effect of TGF-β treatment in spheroids, including investigating the effect on known downstream targets of TGF-β, will provide interesting insight as to how TGF-β is affecting spheroid morphogenesis.

It was noted that an induction of EMT in HCC spheroids is accompanied by a reduction of acinar morphogenesis, suggesting that acinar morphogenesis and formation of protruding spheroids represent two opposite ends of the spectrum of epithelial and mesenchymal conversion, indicating that acinar morphogenesis represents a polarized, well-differentiated epithelial-like structure, whereas protruding spheroids represents a mesenchymal phenotype, with non-acini spheroids representing the middle of the spectrum. Consistently, our orthotopic HCC experiments indicate that HCC cells cultured in 3D are more highly tumorigenic and metastatic than cells in 2D culture. Furthermore, HCC cells from 3D spheroids could induce larger and more aggressive tumors than 2D culture cells when the same numbers of cells were inoculated in mice livers, and TGF-β could accelerate these processes by inducing metastases. Our results indicate that TGF-β enhances cell reprogramming by accelerating EMT, which is more pronounced in an organotypic culture model than in a conventional 2D-culture model. Taken together, we suggest that this 3D model is a reliable *in vitro* system to effectively assess HCC aggressiveness and its underlying molecular mechanisms, and also may prove to be a useful system to be adapted for the culture of primary cells from HCC resected patient samples for the examination of chemotherapeutic interventions, such as sorafenib, for personalized drug treatment. The lack of primary HCC cells in this 3D culture model is one of the main deficiencies of this study. Thus, this 3D culture system will be very important for a number of future studies including those with patient-derived HCC cells and those that examine the mechanisms of drug resistance in these spheroids, including determining if there is a correlation between EpCAM and drug resistance, and the mechanism of drug resistance, for example if there is a decreased uptake of drug into the spheroids. It will also be very interesting to further investigate the polarization of spheroids formed in AlgiMatrix culture, and to characterize proteins transporters that are localized to the transmembrane, which could also confer resistance to chemotherapeutic treatment. Finally, by assessing the degree of protruding cell sphere formation or acinar morphogenesis, this method may be useful to effectively test various candidate driver genes and screen small molecule inhibitors that block this process in a high throughput fashion.

## Materials and Methods

### Cell lines, plasmids and chemicals

For both 2D and 3D culture, all cell lines were cultured with the specific media that has been established for each cell line. HuH1, HuH7 and MHCC97-H cells were cultured in Dulbecco’s modified Eagle Medium (Life Technologies, Grand Island, NY) supplemented with 10% fetal bovine serum (FBS), penicillin, streptomycin and L-glutamine (PSG). HepG2, Hep3B and SK-Hep-1 cells were cultured in Minimum Essential Medium (Life Technologies) supplemented with 10% FBS, PSG, non-essential amino acids and sodium pyruvate. M50 Super 8× TOPFlash and M51 Super 8× FOPFlash were purchased from Addgene (Cambridge, MA). pLKO.1-EpCAM shRNA and pLKO.1-eGFP shRNA lentiviral vectors were purchased from GE Healthcare (Lafayette, CO). R980-M19-663, a lentiviral vector with CMV13 promoter-driven firefly luciferase and eGFP expression was purchased from Frederick National Laboratory for Cancer Research (Frederick, MD). Lentiviral transduction was performed using Trans-Lentiviral shRNA Packaging System (GE Healthcare) or Lenti-vpak packaging kit (Origene, Rockville, MD). Fiduxosin was purchased from Sigma-Aldrich (St. Louis. MO). AV-606 was kindly provided by Avalon Pharmaceuticals (Germantown, MD). TGF-β1 was purchased from Peprotech (Rocky Hill, NJ).

### Organotypic culture

AlgiMatrix^TM^ 3D Culture System (6-well plate) (Life Technologies) was used for the organotypic cell culture. One million cells were seeded to each well and cultured in the complete cell culture medium. For the examination of the sphere histological structure, paraffin-embedded spheres were sectioned and stained with haematoxylin and eosin. For collecting spheres, the matrix was dissolved using AlgiMatrix Dissolving Buffer (Life Technologies) and spheres were pelleted by centrifugation at 300 g for 5 min. For counting the number of spheres, they were suspended with complete cell culture medium and seeded onto a 6-well plate. The number of spheres attached to the plate was counted using a microscope (IX51 Inverted Microscope, Olympus, Tokyo, Japan) from triplicate determinations.

### Immunocytochemistry

Collected spheres were seeded to 8-well chamber slides (Thermo Fisher Scientific, Waltham, MA) and fixed with 4% paraformaldehyde. Immunofluorescence staining was performed using anti-E-cadherin (Cell Signaling Technology, Danvers, MA), anti-EpCAM (DAKO, Glostrup, Denmark), anti-active caspase-3 (Abcam, Cambridge, MA), anti-vimentin (Abcam) antibodies. Alexa 488- or Alexa 568-conjugated goat anti-rabbit and anti-mouse antibodies (Life Technologies) were used as secondary antibodies. Images were taken with a LSM 710 Confocal Microscope (Carl Zeiss, Jena, Germany).

### Western blotting

Protein lysates were separated on 4–12% SDS-polyacrylamide gels (Life Technologies) and transferred to a nitrocellulose membrane (Life Technologies). Protein detection was performed using an anti-EpCAM monoclonal antibody (R&D Systems, Inc., Minneapolis, MN), anti-β-actin monoclonal antibody (Sigma-Aldrich), anti-E-cadherin monoclonal antibody (Cell Signaling Technology), anti-vimentin monoclonal antibody (Abcam), anti-phospho-Smad2 monoclonal antibody (Cell Signaling), and anti-Cyclin D1 monoclonal antibody (Cell Signaling).

### Luciferase reporter assay

Lipofectamine 2000 (Life Technologies) was used to cotransfect cells with M50 Super 8× TOPFlash or M51 Super 8× FOPFlash, and pRL-null *Renilla* luciferase plasmid which was used as an internal control. Firefly and Renilla luciferase activities were determined using a Dual-Luciferase Reporter Assay System (Promega, Madison, WI). For luciferase activity in 3D culture, cells were first plated in 2D and transfected with reporter plasmids as above. Cells were then resuspended as a single cell solution and seeded into AlgiMatrix with each drug or DMSO control. Spheroids were collected after 7 days and luciferase activity was measured. Relative luciferase activities were calculated and compared between the samples.

### TGF-β Treatment

For immunofluorescence experiments, 2.5 ng/ml TGF-β was added to AlgiMatrix culture at day 5, and spheres were harvested at day 7. For Western blot analysis, 5 ng/ml TGF-β was added to AlgiMatrix culture at day 3, and spheres were harvested at day 7. For *in vitro* evaluation of TGF-β effect on spheroids, 2.5 or 5 ng/ml TGF-β was added to AlgiMatrix culture at day 5, and spheres were harvested at day 7. For *in vivo* experiments, 2D cells were treated with 2.5 ng/ml TGF-β for 12 hours prior to harvesting and injection. 3D spheres were treated with 2.5 ng/ml TGF-β for 12 hours on day 5 of AlgiMatrix culture and immediately harvested prior to injection.

### 3D invasion assay

Spheres were collected from AlgiMatrix scaffold at Day3 and seeded to a 96-well plate coated with 50 ul Cultrex ^®^Spheroid Invasion Matrix (Trevigen, Gaithersburg, MD). After incubation at 37 °C for 2 days with 5 ng/ml TGF-β, the number of spheres was counted using a microscope from triplicate determinations.

### Animal Study

Four-week-old NOD/SCID mice (NOD.CB17-*Prkdc*^*scid*^ /NcrCrl) were purchased from Charles River Laboratories, Inc. (Wilmington, MA). The animal study protocol was approved by the National Cancer Institute-Bethesda Animal Care and Use Committee. The experimental protocols were carried out in accordance with the appropriate NIH biosafety guidelines for research involving biological materials. Cells were stably transfected with luciferase and GFP expression vectors. Immediately prior to injection, an aliquot of 3D spheres was taken, spheres were dissociated, and cell number was counted to quantify cell number in 3D spheres, in order to ensure that an equal number of cells from 2D or 3D culture injected into each animal. Cells were suspended with Hank’s Balanced Salt Solution (1 × 10^6^ cells/50 μl) and injected into the left hepatic lobe as previously described^29^. Every 2 weeks, luciferase signals were measured using IVIS Lumina Series III (PerkinElmer, Waltham, MA) after intraperitoneal injection of 3 mg D-Luciferin (PerkinElmer). Luciferase signals were quantified using Living Image Software (PerkinElmer). Mice were sacrificed 4 weeks after the injection and the number of nodules formed in the liver was counted. The tumors and metastatic nodules of the peritoneum and diaphragm were fixed with 10% formalin, followed by embedding with paraffin.

### Histology and immunohistochemistry

The paraffin blocks were sectioned at 5μm and stained with haematoxylin and eosin. Anti-HLA A antibody (Abcam) and Envision^TM^+ Kit (Dako, Glostrup, Denmark) were used for detecting HLA-A.

### Statistical analysis

Statistical analysis was performed using un-paired two-tailed student’s t-tests, with a significance level of p < 0.05. To compare variances, a F-test was performed for each analysis and Welch’s correction was applied when variances were significantly different. GraphPad Prism software was used for all statistical calculations.

## Additional Information

**How to cite this article**: Takai, A. *et al.* Three-dimensional Organotypic Culture Models of Human Hepatocellular Carcinoma. *Sci. Rep.* **6**, 21174; doi: 10.1038/srep21174 (2016).

## Supplementary Material

Supplementary Information
