# Supplementary material for: Three-dimensional Organotypic Culture Models of Human Hepatocellular Carcinoma
Source: Sci Rep. 2016 Feb 16;6:21174. doi: 10.1038/srep21174 (PMC4754778; doi:10.1038/srep21174)
Supplement: Supplementary Information [file srep21174-s1.pdf]

Three-dimensional Organotypic Culture Models of Human Hepatocellular Carcinoma

Atsushi Takai[1], Valerie Fako[1], Hien Dang[1], Marshonna Forgues[1], Zhipeng Yu[1], Anuradha Budhu[1], Xin Wei Wang[1,*]

[1]Laboratory of Human Carcinogenesis, Center for Cancer Research, National Cancer Institute, Bethesda, Maryland 20892, USA

*Correspondence: Xin Wei Wang, National Cancer Institute, 37 Convent Drive, Building 37, Room 3044A, Bethesda, Maryland 20892; Email: xw3u@nih.gov; Phone: 301-496-2099

Running title: A three-dimensional organotypic cell culture model exemplifies molecular features of hepatocellular carcinoma

Supplementary information

Supplemental Figure legends

Supplemental Figure 1. Representative images of Huh1 spheres cultured in each matrix examined. Black arrows point to examples of sphere fusion. Scale bars are 50μm.

Supplemental Figure 2. Representative images of spheres of various cell lines grown in AlgiMatrix. All the spheres were collected at Day 7 and stained with H&E. Scale bars are 50μm.

Supplemental Figure 3. Representative images of Huh-1 spheres with EpCAM shRNA. All the spheres were collected at Day 7 and stained with E-cadherin (green) and EpCAM (red). Scale bars are 50μm.

Supplemental Figure 4. Relative number of Huh1 spheres (white bars) and MHCC97-H spheres (black bars) treated with DMSO, AV-606 and fiduxosin are shown.

Supplemental Figure 5. Relative number of Huh1 spheres harvested at Day 7, treated with TGFβ beginning at Day 1 of AlgiMatrix culture, is shown.

Supplemental Figure 1

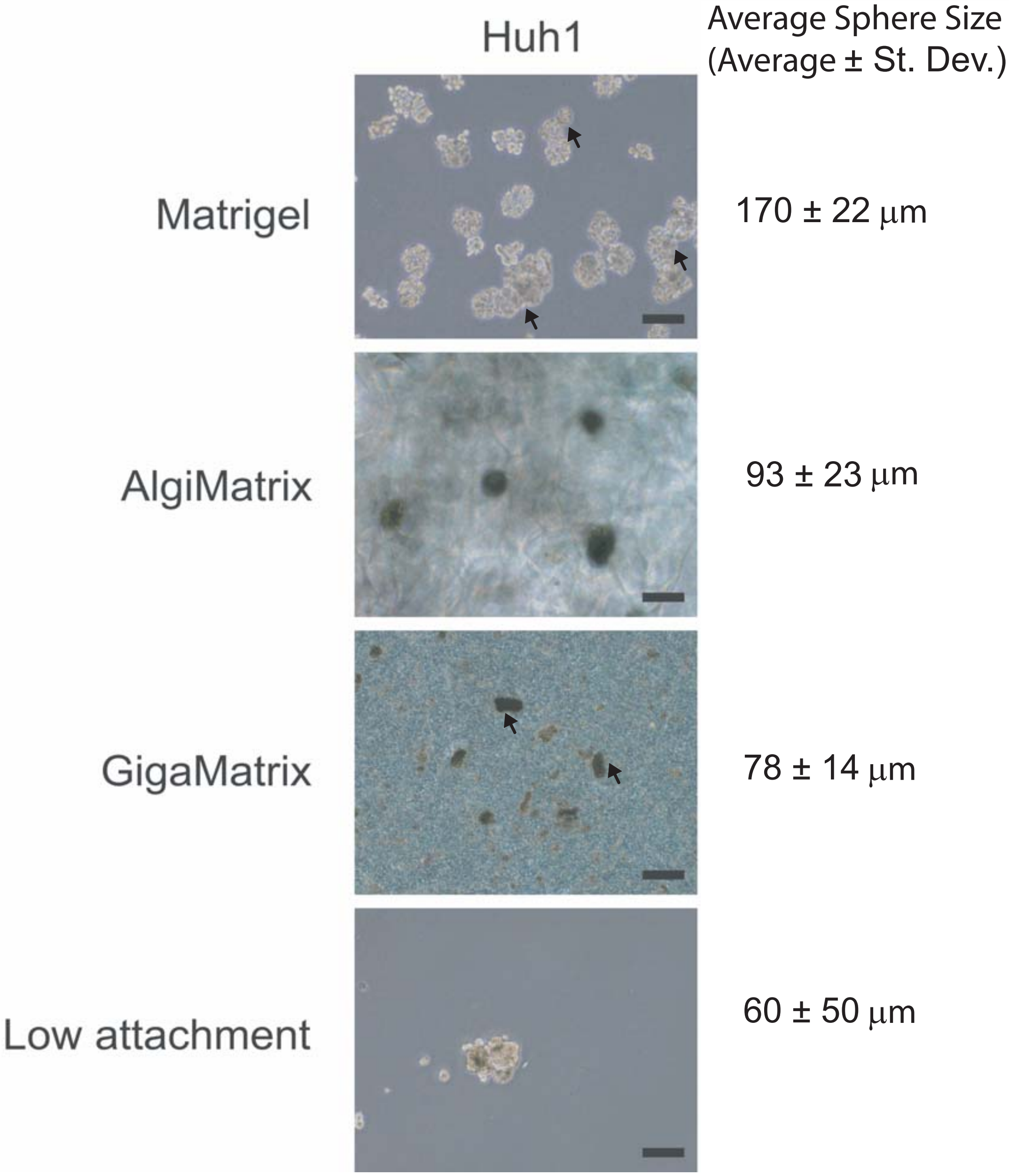

Supplemental Figure 2

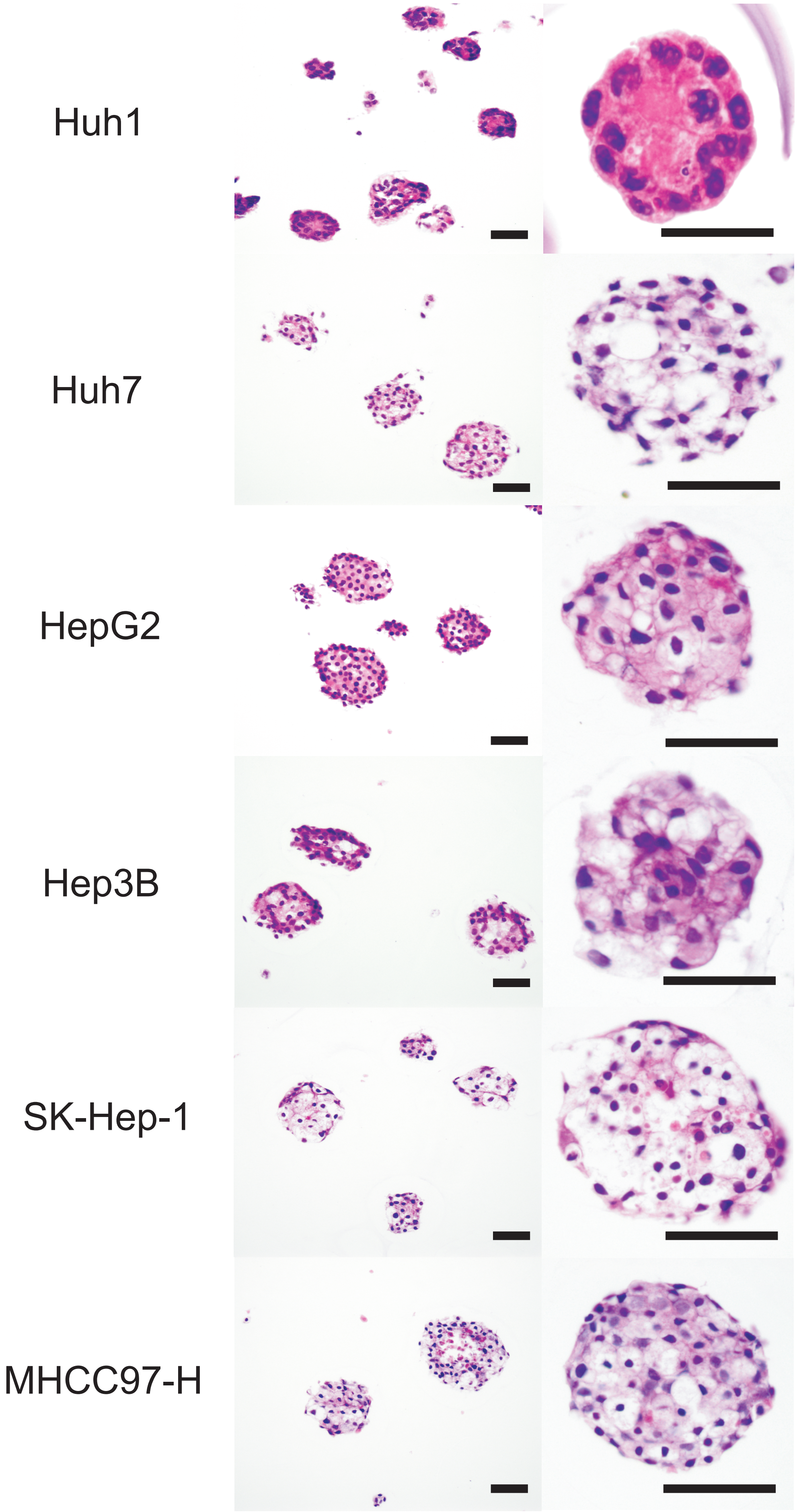

Supplemental Figure 3

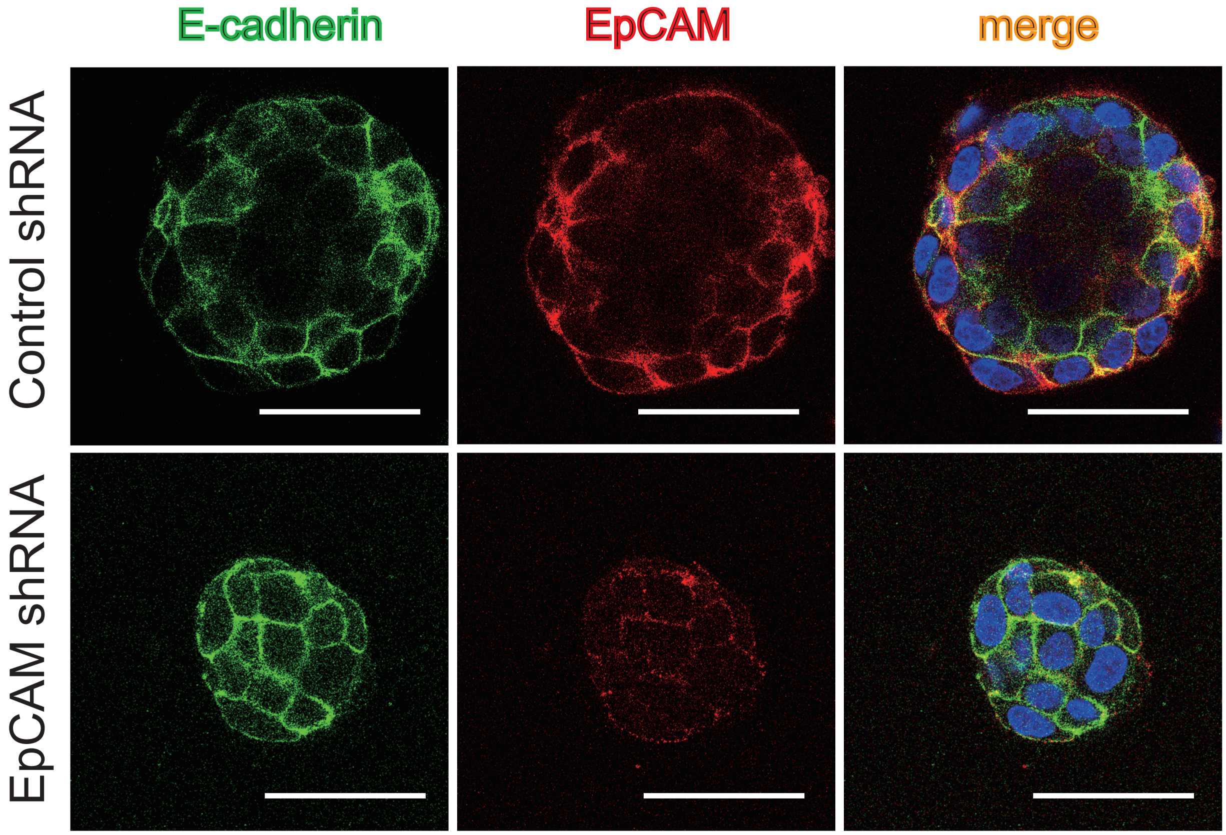

Supplemental Figure 4

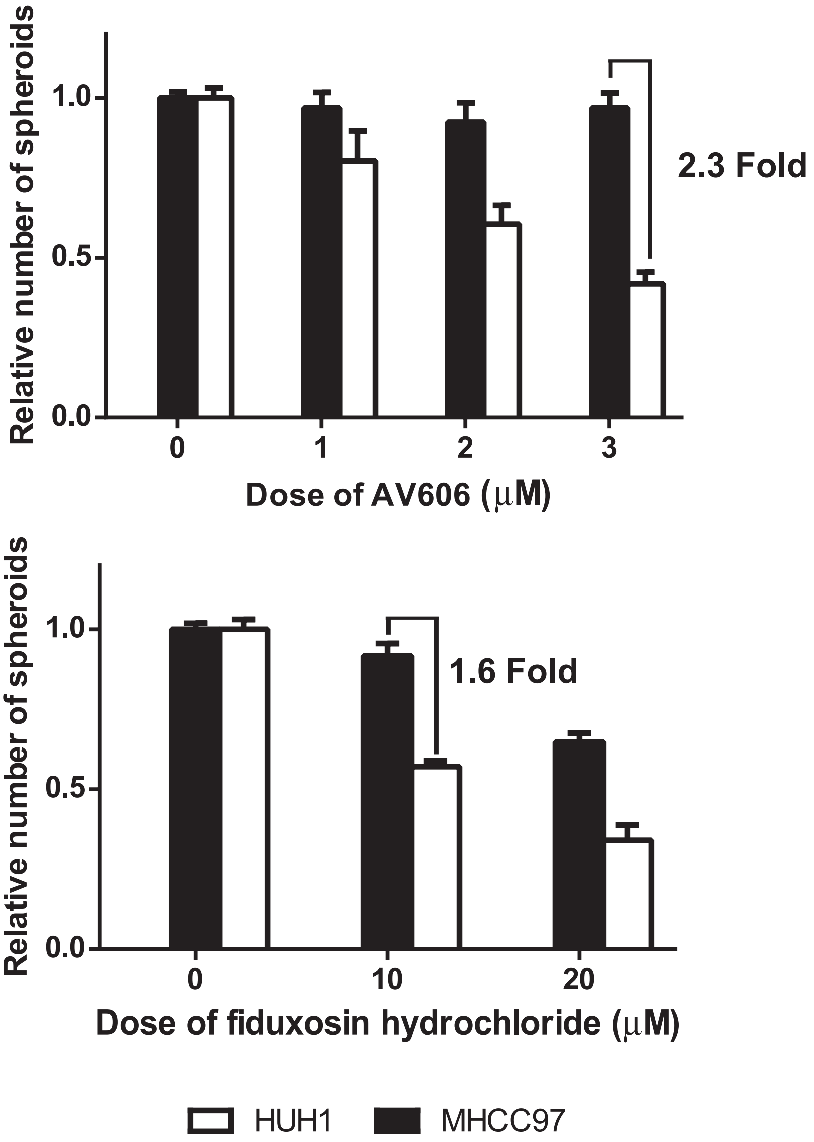

Supplemental Figure 5

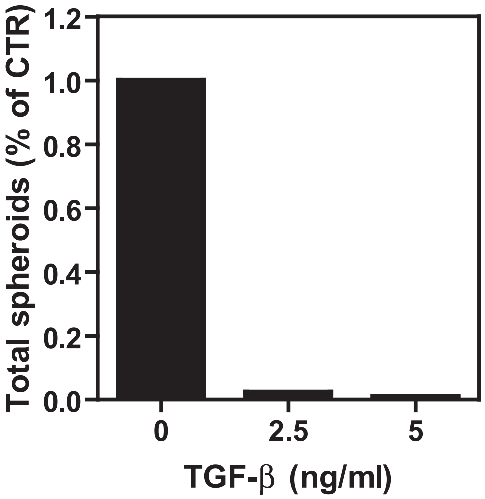

Supplemental Table 1. Summary of characteristics of each culture system

	Size Range Observed	Morphology Observed	Sphere Fusion Observed	Ease in Sphere Collection
**AlgiMatrix**	60-240 µm	Round	No	Easy
**Matrigel**	50-200 µm	Round/Irregular	Yes	Easy
**GigaMatrix**	50-125 µm	Irregular	Yes	Difficult
**Ultra-Low Attachment**	20-300 µm	Round	No	Easy

Supplemental Table 2. Frequencies of HCC and metastasis in orthotopic liver cancer model

	No treatment (3D) (n=9)	TGF treatment (3D) (n=10)	No treatment (2D) (n=9)	TGF treatment (2D) (n=7)
**Number of mice with HCC (%)**	8 (89)	9 (90)	6 (67)	6 (86)
**Peritoneal metastasis (%)**	0 (0)	1 (10)	0 (0)	0 (0)
**Diaphragm metastasis (%)**	0 (0)	1 (10)	0 (0)	0 (0)
